# Exposure to and experiences with a computerized decision support intervention in primary care: results from a process evaluation

**DOI:** 10.1186/s12875-015-0364-0

**Published:** 2015-10-16

**Authors:** Marjolein Lugtenberg, Dennis Pasveer, Trudy van der Weijden, Gert P. Westert, Rudolf B. Kool

**Affiliations:** Scientific Institute for Quality of Healthcare (IQ healthcare), Radboud university medical center, P.O. Box 9101, 6500 HB Nijmegen, The Netherlands; Scientific Center for Care and Welfare (Tranzo), Tilburg School of Social and Behavioral Sciences, Tilburg University, P.O. Box 90153, 5000 LE Tilburg, The Netherlands; School for Public Health and Primary Care (CAPHRI), Department of General Practice, Maastricht University, P.O. Box 616, 6200 MD Maastricht, The Netherlands

**Keywords:** Clinical decision support, Clinical practice guidelines, Primary care, Process evaluation, Barriers

## Abstract

**Background:**

Trials evaluating the effects of interventions usually provide little insight into the factors responsible for (lack of) changes in desired outcomes. A process evaluation alongside a trial can shed light on the mechanisms responsible for the outcomes of a trial. The aim of this study was to investigate exposure to and experiences with a computerized decision support system (CDSS) intervention, in order to gain insight into the intervention’s impact and to provide suggestions for improvement.

**Methods:**

A process evaluation was conducted as part of a large-scale cluster-randomized controlled trial investigating the effects of the CDSS NHGDoc on quality of care. Data on exposure to and experiences with the intervention were collected during the trial period among participants in both the intervention and control group - whenever applicable - by means of the NHGDoc server and an electronic questionnaire. Multiple data were analyzed using descriptive statistics.

**Results:**

Ninety-nine percent (*n* = 229) of the included practices generated data for the NHGDoc server and 50 % (*n* = 116) responded to the questionnaire: both general practitioners (GPs; *n* = 112; 49 %) and practice nurses (PNs; *n* = 52; 37 %) participated. The actual exposure to the NHGDoc system and specific heart failure module was limited with 52 % of the GPs and 42 % of the PNs reporting to either never or rarely use the system. Overall, users had a positive attitude towards CDSSs. The most perceived barriers to using NHGDoc were a lack of learning capacity of the system, the additional time and work it requires to use the CDSS, irrelevant alerts, too high intensity of alerts and insufficient knowledge regarding the system.

**Conclusions:**

Several types of barriers may have negatively affected the impact of the intervention. Although users are generally positive about CDSSs, a large share of them is insufficiently aware of the functions of NHGDoc and, finds the decision support not always useful or relevant and difficult to integrate into daily practice. In designing CDSS interventions we suggest to more intensely involve the end-users and increase the system’s flexibility and learning capacity. To improve implementation a proper introduction of a CDSS among its target group including adequate training is advocated.

**Trial registration:**

Clinical trials NCT01773057.

**Electronic supplementary material:**

The online version of this article (doi:10.1186/s12875-015-0364-0) contains supplementary material, which is available to authorized users.

## Background

Computerized decision support systems (CDSSs) have the potential to improve quality of care [1, 2]. To the extent that their content is guideline-based, they can support physicians in adhering to the best-available evidence as presented in clinical guidelines, and ultimately lead to improved patient outcomes. Evidence on their uptake and effectiveness, however, is thus far limited and is usually retrieved from small-scale academic-driven settings, in which CDSSs aimed at a limited number of specific decision points are tested [3–7].

In the Netherlands, a CDSS for primary care - NHGDoc - was developed in 2006, as a collaborative effort between the Dutch college of General Practitioners (NHG) [8] and ExpertDoc BV [9], a private company. NHGDoc covers multiple domains of care and provides a variety of patient-specific advices during patient consultation for both general practitioners (GPs) and practice nurses (PNs). Its content is based on the NHG guidelines, the national prevailing guidelines for general practice. The system is gradually being implemented at a large scale and is currently available to approximately 65 % of all Dutch general practices [9].

To evaluate the uptake of NHGDoc and its effectiveness in terms of improving quality of primary care, a large-scale cluster randomised controlled trial (RCT) was conducted within 231 general practices in the Netherlands. More specifically, this two-arm cluster RCT with a follow-up period of 1 year, assessed the effectiveness of NHGDoc with respect to improving the process of medical care (e.g. prescription behavior) as well as patient outcomes (e.g. hospital admissions), which is described in detail in the Methods section [10]. Preliminary analyses reveal that no effects were found on the identified outcome measures [11].

Delivering a complex intervention such as a CDSS in a trial setting does not guarantee that the target group is actually exposed to the intervention as planned. Particularly, in cluster RCTs exposure to the intervention may vary considerably between participating practices [12]. Also, participants’ experiences with the intervention may affect exposure and may help explain the results of a trial. A process evaluation can shed light on the factors/mechanisms responsible for the outcomes of a trial [12, 13].

Therefore, we conducted a process evaluation alongside the trial. The aim of this study was to investigate the exposure to and experiences with our CDSS quality improvement intervention, in order to gain insight into the factors contributing to the intervention’s impact. Results of the process evaluation can provide insight in the feasibility and reproducibility of the intervention [14] and can be used to improve the implementation of NHGDoc as well as similar CDSS interventions.

## Methods

### Description of the intervention

#### The CDSS NHGDoc

NHGDoc is a CDSS integrated within the electronic health record system (EHRS) and based on the NHG guidelines, the prevailing guidelines for general practice in the Netherlands [8]. It provides GPs and PNs evidence-based and patient-specific advices during consultation in terms of patient data registration, drug prescription and management.

##### Basic functions of NHGDoc

When the GP or PN opens a patient file in the EHRS, anonymous patient and performance data are sent to the NHGDoc server. The patient and performance data are compared to the digitized guideline recommendations and in case of a discrepancy between current and advised care, an alert will be sent back to the GP or PN. Also, users have the option to ask or provide feedback from/to ExpertDoc, the organization that has developed and maintains NHGDoc, about the received alert. See [15] for a detailed description of the NHGDoc system.

##### Personalization functions of NHGDoc

Aside from the basic functions (alerts and feedback), NHGDoc allows the user to adapt the decision support to meet their personal preferences in two different ways. By using *alert settings*, users can adjust the preferences of the alerts to match their personal needs. They can choose to switch alerts on and off on demand at several levels: the system, the modules (NHGDoc domains), the types of alerts (patient data registration, management, drug prescription), and the patients. The *reporting settings* allow users to request specific reports with respect to the number and types of alerts they have received per domain within a specific period of time (per year, per month, per week of per day). See [15] for a detailed description of the NHGDoc system.

To evaluate the effects of NHGDoc on quality of care we conducted a cluster randomized controlled trial [10]. All general practices in the Netherlands that, at the time of recruitment had NHGDoc at their disposal, were invited to participate in the study (*n* = approx. 1.100). Our power analysis revealed that we needed to include at least 122 general practices to detect an effect on our main primary outcome measures. A comprehensive recruitment plan was conducted, consisting of a combination of direct invitations via postal mailings as well as emails, combined with announcements of the evaluation study in several relevant Dutch journals, websites, newsletters and through social media (i.e. Facebook, Twitter and LinkedIn) [10]. A total of 231 general practices gave their consent to participate in the trial. 115 practices were randomly assigned to the control arm, whereas 116 practices were allocated to the intervention arm.

#### Regular NHGDoc modules (control arm)

General practices assigned to the control arm received the regular NHGDoc decision support [10]. These practices received decision support with respect to all modules (NHG guidelines) that – at the onset of the trial – had already been integrated into NHGDoc. These were cardiovascular risk management, asthma/COPD, diabetes mellitus type II, thyroid disorders, viral hepatitis and other liver diseases, atrial fibrillation and subfertility.

#### The NHGDoc heart failure module (intervention arm)

General practices allocated to the intervention arm received the same decision support modules as the control arm, extended with the NHGDoc module on heart failure [10]. The NHGDoc module on heart failure is directly derived from the NHG guideline on heart failure [16]. It consists of three types of alerts:

1. Alerts on heart failure in terms of registering patient data;
2. Alerts on heart failure in terms of prescribing (or adjusting the dose of) drugs;
3. Alerts on heart failure in terms of (paying attention to) management aspects.
  See [15] for a detailed description of the module.

At the onset of the trial the NHGDoc heart failure module was activated in the intervention group. Other than that, no activities and time investments were required. General practices and the staff working within these practices were blinded to group allocation. They were even unaware of participating in an intervention trial with a concurrent control group. The trial did not show a significant effect on the identified primary outcome measures (prescribing of ACE inhibitors/angiotensin II; prescribing of beta-blocker; prescribing of diuretics) [11].

### Data collection and study measures

The need for ethical approval for the NHGDoc evaluation study was waived by the research ethics committee of the Radboud university medical center. Data for the process evaluation were collected among all participating practices in both the intervention (*n* = 115) and control arm (*n* = 116) - whenever applicable - in two different ways: by collecting data from the NHGDoc server and by conducting a questionnaire. Informed written consent was obtained from all practices: each general practice filled out an online registration form in which they agreed with the design of the study and the use of NHGDoc server data as described on the website (www.nhgdoc-evaluatie.nl) and gave consent on behalf of all practice staff (GPs and PNs). Responding to the questionnaire was voluntary and again information was provided on handling the data in terms of confidentiality and anonymity.

We used the process evaluation framework as described by Hulscher et al. [12] whenever applicable. Besides from describing the intervention itself, this framework recommends to describe the *actual exposure to the intervention* as well as the *experiences* with the interventions [12].

#### NHGDoc server

We extracted the following data from the NHGDoc server to measure *exposure* to the intervention in both study groups: NHGDoc activity (send NHGDoc requests), switched off NHGDoc modules including heart failure (intervention group only), opening of alerts including for heart failure patients (intervention group only). Data were collected continuously during the trial and were sent to our research institute (IQ healthcare) on a monthly base [10].

#### Questionnaire

At the end of the trial an electronic questionnaire (see Additional file 1) was sent to all participating practices (*n* = 231). From each general practice we invited one GP and one PN (if applicable) to fill out the questionnaire. Aside from demographic and professional characteristics (e.g., age, gender, number of hours worked weekly), *exposure* to and *experiences* with the CDSS intervention were measured.

*Exposure* was measured with six statements about the level of use of the NHGDoc system as a whole; the alert function; the feedback function; the My NHGDoc function, and more specifically the alerts settings and reporting settings. Responses were rated on a 5-point Likert scale (ranging from 1. not at all to 5. very much).

*Experiences* with the intervention were measured in two different ways: by measuring attitudes towards CDSSs in general and by measuring perceived barriers and suggested interventions to using NHGDoc. Prior to developing the questionnaire, we conducted a qualitative focus group study to identify the range of barriers that GPs and PNs perceive in using NHGDoc or similar CDSSs in practice. Three focus group sessions were conducted in which 24 primary care practitioners (PCPs) participated (general practitioners, general practitioners in training and practice nurses), varying from 7 to 9 per session. In each focus group, barriers to using CDSSs were discussed using a semi-structured literature-based topic list. Two researchers independently performed thematic content analysis using the software program Atlas.ti 7.0.

This resulted in a framework of barriers, which is described elsewhere in detail [15]. In general, three groups of barriers emerged, related to 1) the users’ knowledge of the system, 2) the users’ evaluation of features of the system (source and content, output, and functionality), and 3) the interaction of the system with external factors (patient-related and environmental factors). Whereas the focus group study was conducted to identity all relevant barriers to using CDSSs, the survey study aimed to validate and quantify these findings.

Eight statements were included to measure the attitudes towards CDSSs in general, based on literature on attitudes towards using clinical practice guidelines [17, 18], complemented with results from the focus group study [15]. A 5-point Likert scale was used to rate the extent of agreement with the statements (ranging from 1. strongly disagree to 5. strongly agree).

To measure the perceived barriers three groups of barriers were included, based on the framework that emerged from the focus group study [15].

- *Knowledge*-*related barriers* were measured with six statements referring to knowledge about the (specific functions of the) system (e.g. ‘I am aware of the option to ask for or to provide feedback in NHGDoc’).
- *Barriers related to the evaluation of the features of the CDSS* were measured with 11 statements (e.g. ‘I believe the loading of alerts takes too long’).
- *External barriers interacting with the CDSS* were measured with 6 statements (e.g. ‘Using NHGDoc has a negative effect on patient-doctor communication’).

The knowledge-related barriers were scored on a 2-point scale (yes/no). All other statements were rated a 5-point scale (ranging from 1. strongly disagree to 5. strongly agree), with the option ‘not applicable’ (6.) added to the response scale. Suggested interventions to improve CDSS usage were also discussed and will be described elsewhere.

Finally, the intended ‘blinding’ in terms of the topic of the intervention was checked among the participants by formulating the following open question ‘Do you have any idea on which clinical topic this study focused on?’.

### Data analysis

Data from the NHGDoc server measuring exposure to NHGDoc were analyzed using descriptive statistics. To analyze exposure as measured by the questionnaire we categorized the scores 1 and 2 to indicate a low level of use, we coded 3 as a moderate level of use, and combined the scores 4 and 5 to reflect an intermediate to high level of use.

To analyze the attitudes towards CDSSs in general we categorized the scores 4 and 5 (agree/strongly agree) indicating agreement; the scores 3 to refer to a neutral attitude, and the scores 1 and 2 (strongly disagree/disagree) to indicate disagreement.

The knowledge-related barriers were calculated by the percentage of respondents that indicated not to be aware of NHGDoc or its specific functions (score 1). For the second and third group of barriers (attitude-related and external barriers) the barrier statements that were stated positively rather than negatively, were first recoded, so that a higher score indicated a higher level of perceived barriers. Next, the scores 4 and 5 were combined to indicate the percentage users that indicate a barrier to be applicable.

## Results

### Description of study sample

The 231 participating practices were an acceptable reflection of the population of Dutch GP practices, with solo practices only being slightly overrepresented [19].

#### Description of sample NHGDoc server

Almost all recruited practices (229/231 = 99 %) generated data into the NHGDoc server database; two were eliminated from the trial due to technical problems. Because of this high rate, the background characteristics are comparable to the study population.

#### Description of sample of questionnaire

Of the 231 practices 50 % responded to the survey (see Table 1). The responding practices were equally distributed between the intervention group and control group. Most practices concerned solo practices (57 %) and used the EHRS MicroHIS X (56 %), which is comparable to our study population (resp. 56 % and 58 %).

**Table 1:** Characteristics of the responding GPs and PNs and their practices compared to the total study population

	Respondents *N*	%	Total study population (%)
General practitioners (*N* = 231^a^)	112	48.5	(*N* = 537)
Sex			
Male	77	68.8	52.7
Female	35	31.3	47.3
Practice nurses (*N* = 141^b^)	52	36.9	(*N* = 225)
Sex			
Male	1	1.9	6.7
Female	51	98.1	93.3
Practices (*N* = 231^c^)	116	50.2	(*N* = 231)
Group			
Control group	58	50.0	50.0
Intervention group	58	50.0	50.0
Type of EHRS			
MicroHIS X	65	56.0	55.8
Promedico-ASP	51	44.0	43.3
Type of practice			
Solo	66	56.9	57.6
Duo	32	27.6	26.4
Group (>2)	18	15.5	15.2

*GPs* general practitioners

*PNs* practice nurses

^a^Total number of GPs that were send a survey. Although there were more GPs in most practices, we only invited one GP per practice to participate in the survey

^b^Total number of PNs that were send a survey based on the number of practices that had reported to have at least one practice nurse employed

^c^Total number of practices that were send a survey to at least one GP/PN

From the group of responding GPs (49 %) relatively more men (69 %) were included compared to our total study population (69 vs. 53 %). The majority of the participating GPs were aged between 55 and 64 years (46 %) (no comparison possible). Ninety-eight percent of the responding PNs (37 %) were women (98 %), a little more compared to our study population (93 %). Most of them were aged between 45 and 54 years of age (no comparison possible).

### Exposure to the CDSS intervention

#### NHGDoc server

**Fig. 1:**
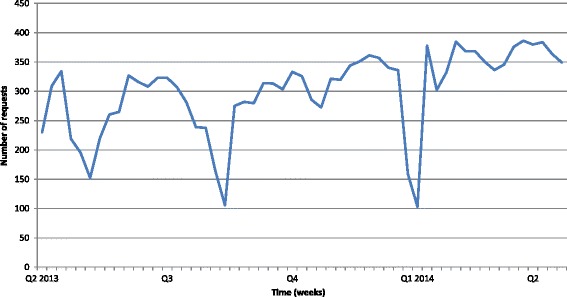
NHGDoc activity of the participating practices during the year of the trial. *Q* quarter

Figure 1 indicates the level of NHGDoc activity in participating practices illustrated by the number of requests sent per week. As can be seen, there are three large negative spikes during the trial year.

The NHGDoc alerts concerning heart failure patients were opened in only 0.24 % of the cases. This was 0.35 % for the total patient population. No trend towards increased use was detected during the trial period.

Data from the NHGDoc server also revealed that less than 2 % of the participating primary care providers used the personalization functions of NHGDoc to switch off one or more NHGDoc domains. With respect to the specific Heart failure domain this percentage was less than 1 %.

#### Questionnaire

Table 2 shows the reported use of the system NHGDoc and its specific functions, as identified by the questionnaire. As can be seen contrasting subgroups can be identified: approximately half of the GPs (52 %) and 42 % of the PNs reported to either never or rarely use the system whereas almost 10 % of the GPs and 20 % of the PNs use it very often or all the time.

**Table 2:** Reported use of NHGDoc and its specific functions

	Never/rarely			Sometimes			Often/always		
	GP	PN	Total	GP	PN	Total	GP	PN	Total
NHGDoc system	58 (52 %)	21 (42 %)	79 (49 %)	42 (38 %)	19 (38 %)	61 (38 %)	11 (10 %)	10 (20 %)	21 (13 %)
Alert function	57 (51 %)	19 (40 %)	76 (48 %)	42 (38 %)	18 (38 %)	60 (38 %)	12 (11 %)	11 (23 %)	23 (14 %)
Feedback function	97 (87 %)	42 (86 %)	139 (87 %)	12 (11 %)	6 (12 %)	18 (11 %)	2 (2 %)	1 (2 %)	3 (2 %)
My NHGDoc	80 (72 %)	32 (65 %)	112 (70 %)	26 (23 %)	8 (16 %)	34 (21 %)	5 (5 %)	9 (18 %)	14 (9 %)
Alerts settings	90 (81 %)	41 (84 %)	131 (82 %)	14 (13 %)	4 (8 %)	18 (11 %)	7 (6 %)	4 (8 %)	11 (7 %)
Reporting settings	91 (82 %)	45 (92 %)	136 (85 %)	17 (15 %)	2 (4 %)	19 (12 %)	3 (3 %)	2 (4 %)	5 (3 %)

From the basic functions half of the GPs (51 %) and 40 % of the PNs reported never or rarely to use the alert functions. Thirty-eight percent of both groups indicated to use the alert functions sometimes. The feedback function demonstrated a lower level of use with 88 % of the GPs and 86 % of the GPs never or rarely using it. Less than 2 % of both groups indicated to use the feedback option often or all the time.

With respect to the personalization functions of My NHGDoc, the majority of both GPs (72 %) and PNs (65 %) reported to never or rarely use this function. Relatively more PNs as compared to GPs used it often or all the time (18 vs. 5 %). Eighty-two percent of the target group never or rarely used the alert settings; for the reporting settings this figure was 85 %.

### Experiences with the NHGDoc intervention

#### Attitude towards CDSSs in general

**Fig. 2:**
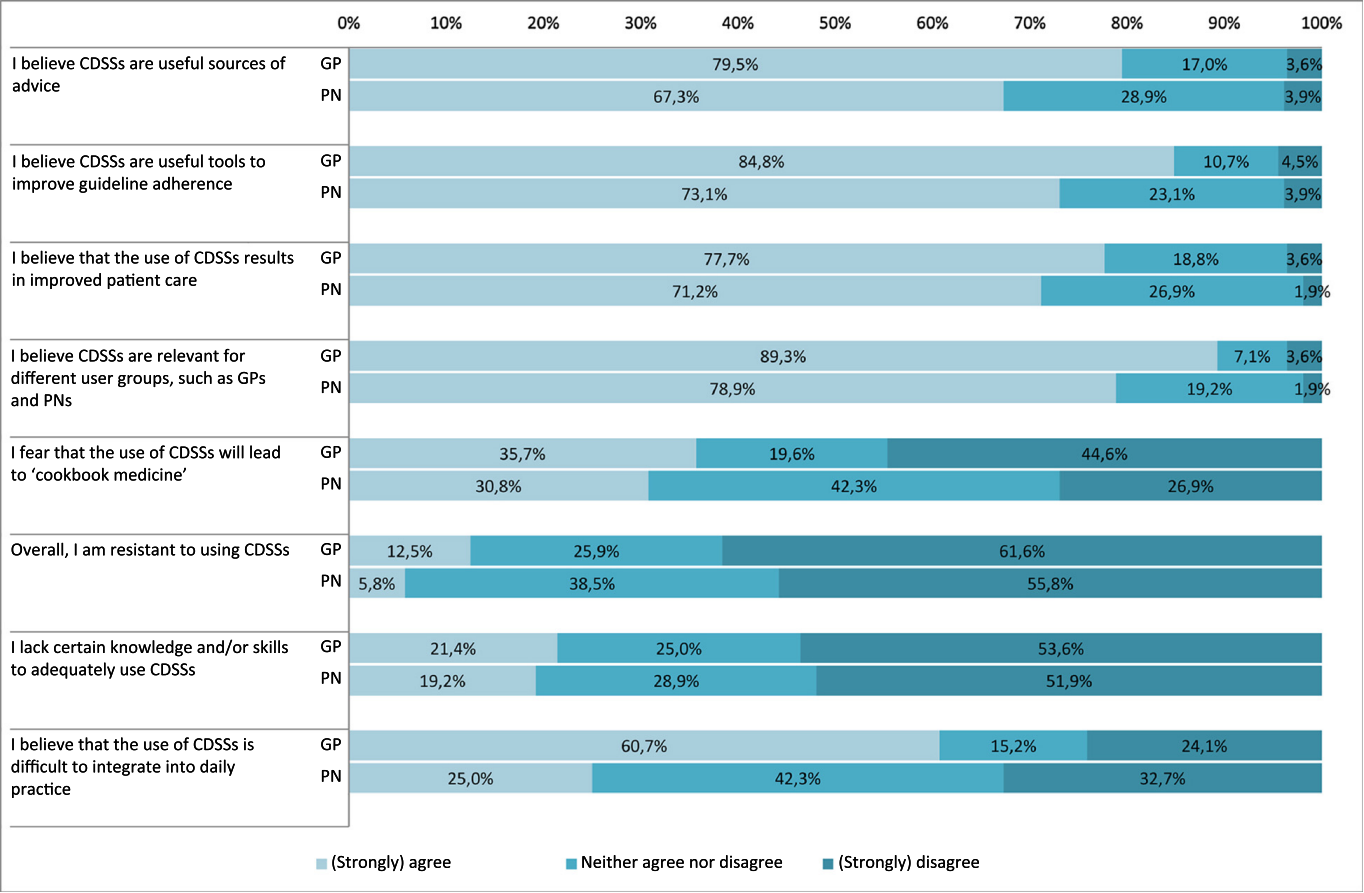
GPs’ (*n* = 112) and PNs’ (*n* = 52) ratings on statements measuring the attitude towards CDSSs in general

As can be seen in Fig. 2, 80 % of the GPs and 67 % of the PNs agreed to the statement that CDSSs are useful sources of advice. In addition, 85 % of the GPs and 73 % of the PNs indicated that they considered CDSSs as useful tools to improve the uptake of guidelines and about three-third of both groups (78 and 71 %) believed that using CDSSs results in improved patient care. Eighty-nine percent of the GPs and 79 % of the PNs believed that CDSSs are relevant for different user groups.

Sixty percent of the GPs and 25 % of the PNs indicated that the use of CDSSs is difficult to integrate in daily practice. About a third of both groups (36 % of GPs and 31 % of PNs) was afraid that using CDSSs leads to ‘cookbook medicine’. One fifth of both groups (21 and 19 %) reported to lack knowledge and skills to properly use CDSSs. Thirteen percent of the GPs and 6 % of the PNs indicated to have overall resistance to working with CDSSs.

#### Experiences with NHGDoc: perceived barriers

Table 3 presents the three groups of barriers as reported by the respondents of the questionnaire.

**Table 3:** Number and percentage of GPs and PNs that perceive barriers to using NHGDoc

	GPs (*N* = 112) *N* (%)	*N* of valid responses	PN (*n* = 52) *N* (%)	*N* of valid responses
Knowledge-related barriers				
Knowledge of existence of NHGDoc	2 (2 %)	111	9 (18 %)	50
Knowledge of basic functions				
Alerts	1 (1 %)	109	0 (0 %)	40
Feedback	45 (41 %)	109	22 (55 %)	40
Knowledge of personalization functions				
My NHGDoc	34 (31 %)	109	16 (40 %)	40
Alerts settings	50 (46 %)	109	26 (65 %)	40
Reporting settings	56 (51 %)	109	28 (70 %)	40
Barriers related to the evaluation of the features of the CDSS				
Source and content of the CDSS				
Reliability of the source of the content	4 (4 %)	104	0 (0 %)	37
Currentness of the content	11 (11 %)	101	6 (16 %)	37
Relevance of the alert content for individual users, with varying needs across time	57 (58 %)	98	24 (62 %)	39
Relevance of the alert content for different user groups	13 (13 %)	99	4 (11 %)	38
Format/lay out of the CDSS content				
Notification method of alerts (too intrusive)	7 (7 %)	105	1 (3 %)	38
Notification method of alerts (uninformative)	17 (17 %)	100	4 (11 %)	37
Readability of the alert text (too wordy/verbose)	15 (15 %)	98	2 (5 %)	38
Functionality of the CDSS				
Responsiveness of the system (retrieval of an alert takes too long)	32 (33 %)	98	14 (38 %)	37
Intensity of alerts (low threshold for triggering alerts)	39 (40 %)	98	12 (32 %)	38
Flexibility (lack of adjustability to personal preferences)	16 (18 %)	90	7 (23 %)	30
Learning capacity of the system (only fixed rules are used)	84 (80 %)	105	27 (75 %)	36
External barriers interacting with the CDSS				
Patient-related factors				
Doctor-patient communication (too much time spent on the computer during consultation)	26 (26 %)	101	8 (22 %)	37
Relevance of alert content for patient (discrepancy between patient’s reason for visit and alert content)	5 (5 %)	100	3 (8 %)	37
Environmental factors				
Limited time available (during and after consultation)	61 (60 %)	101	6 (16 %)	37
Too much additional work required (during and after consultation)	61 (60 %)	100	10 (27 %)	37
Lack of integration with other systems (no direct links to follow-up actions)	25 (27 %)	93	7 (21 %)	34
Fear for misuse of data (patient data and medical practice) by third parties (i.e. health insurers)	11 (11 %)	104	3 (8 %)	36

##### Knowledge-related barriers

As can be seen in Table 3 2 % of the GPs and 18 % of the PNs were not aware of the existence of NHGDoc at all. Of those who did, almost all respondents were aware of the fact that the CDSS sends alerts to users. Fourty-one percent of the GPs and 55 % of the PNs, however, were not aware of the existence of the feedback option. Particularly, a lack of knowledge regarding the option to use the MyNHGDoc personalization functions was found: 31 % of the GPs and 40 % of the PNs were not aware of this function. This particularly applied to the reporting setting options (51 % GPs and 70 % of PNs), but also to the alert setting options (46 % GPs and 65 % of PNs).

##### Barriers related to the evaluation of the features of NHGDoc

From the group of barriers related to the evaluation of the features of the CDSS, particularly the subgroup *functionality* was considered relevant. In both groups a lack of learning capacity of the system (80 and 75 %), a too high intensity of the alerts (40 and 32 %) and a lack of responsiveness of the system (33 and 38 %) were often perceived as barriers. From the subgroup *source and content* of the CDSS the relevance of the alert content for individual users was often perceived as a barrier in both groups (58 and 62 %).

##### External barriers interacting with the CDSS

From the group of external barriers interacting with the CDSS, particularly *environmental factors* were often perceived as barriers. Sixty percent of the GPs indicated the limited time available to be a barrier, whereas only 16 % of the PNs considered this to be a barrier. Also, 60 % of GPs reported that using the CDSS required too much extra work, as compared to 27 % of the PNs. From the *patient*-*related factors*, particularly a negative effect on patient communication was considered as a barrier by 26 % of the GPs and 22 % of the PNs.

#### Blinding

Finally, the blinding check showed that none of the respondents was aware of the fact that heart failure was the focus of the intervention.

## Discussion

This process evaluation revealed that the target group was not exposed to the intervention as planned as the use of NHGDoc and its specific heart failure module was limited. Although (potential) users had a positive attitude towards CDSSs in general, several barriers to using NHGDoc could have hampered the uptake and effectiveness of the intervention. The large share of users that was insufficiently aware of the functions of NHGDoc, as well as the proportion that finds the decision support not useful or not relevant and difficult to integrate into daily practice, indicates that both the CDSS intervention itself as well as its implementation could be improved. These findings are in line with the lack of changes in outcomes of the CDSS intervention.

The actual exposure to NHGDoc and the heart failure module was low, with about half of the (potential) users reporting to either never or rarely use the system and less than 1 % opening alerts. Despite a few negative spikes in NHGDoc activity, which are presumably related to vacation periods of the staff, the activity was quite stable throughout the trial and technical problems did not seem to be a major issue. Also, the low level of exposure to NHGDoc was not related to users deliberately switching of one or more modules of NHGDoc. In interpreting the number of alerts in our study, it is important to realize that the different modules do not result in different alerts each. Rather, the advices are combined in one alert, consisting of an alert window with different tab pages for all relevant domains. Moreover, prior studies have also found high levels of ignoring of alerts among their users [20–22]. Nonetheless, our results suggest that other factors may have prevented our study sample from using NHGDoc as well.

Although the target group of (potential) users had a positive attitude towards CDSSs in general, several barriers were reported that could have affected the uptake and effectiveness of the intervention. The most perceived barriers to using NHGDoc were a lack of learning capacity of the system, the limited time available and the additional work it requires using the CDSS, irrelevant alerts, too high intensity of alerts and insufficient knowledge regarding the system. These findings are consistent with our focus group study and confirm that barriers to using NHGDoc were found within all three main groups of barriers in our previously developed framework of barriers to using CDSSs [15].

With respect to the knowledge-related barriers we found that the target group was insufficiently aware of NHGDoc and its functions, particularly the advanced personalization functions. At the onset of the trial the NHGDoc module on heart failure was activated in the intervention practices, without any further instructions. We deliberately chose for this method, as we aimed at both practitioners and patients being blinded to group allocation, which is important in protecting against bias [23, 24]. Moreover, since all participating practices already had NHGDoc at their disposal, we presumed a certain level of basic knowledge about NHGDoc. However, our findings show a serious lack of knowledge about the system and demonstrate the importance of a proper introduction of a CDSS intervention among its target group. This is particularly challenging when implementing CDSSs at a larger scale, outside the academic setting [15].

Aside from improving the implementation of CDSS interventions among the target group, our results indicate that in our study the CDSS intervention itself also exhibits some shortcomings, often referred to as the ‘intervention failure’ [25, 26]. With regard to the features of the system itself, a perceived lack of learning capacity of the system, irrelevant alerts and a too high intensity of (or a too low of a threshold for) alerts were most often perceived as barriers. It is precisely these types of barriers that often result in ‘alert fatigue’, and the ignoring of alerts [22, 27]. They may be less relevant for the more common CDSSs aimed at a limited number of decision points and may particularly apply in the dynamic and complex primary care setting in which different types of PCPs work with CDSSs covering multiple disease areas [15]. Increasing the system’s flexibility and learning capacity in order to be able to adapt the decision support to meet the varying needs of different users may then become increasingly important.

External barriers interacting with the CDSS were also experienced as barriers which has been found in other studies as well [27–32]. As much as 60 % of the GPs from our sample reported the limited time available and the additional work it requires to use the CDSS as barriers to using it. Obviously, many GPs find it difficult to integrate the use of CDSSs into daily practice. However, we found that these barriers only apply to 16–27 % of the PNs. This is probably related to the nature of their work activities, which contains a larger share of patient data registration, as well as to the way their patient consultation is scheduled. To reduce integration problems it could be helpful to rearrange GPs’ patient consultation as well, with more preparation time before actual patient consultation [15]. Adequate training about how to properly use the system as well as providing the target group with decision support that is tailored to the specific and varying needs of each user, could also contribute in solving these integration problems.

One of the limitations of this study is the seemingly late conduction of this barrier study. However, it should be noted that the NHGDoc system already existed for 5 years at the start of the trial and several small user-satisfaction studies had been conducted among users. Moreover, implementing (CDSS) interventions at a larger scale outside the academic setting, seems to elicit different types of barriers, that cannot be identified in a preceding barrier study among early adopters [15, 33]. Implementing and further developing the system, while conducting prospective formative process evaluations at several stages of implementation, seems therefore necessary. It allows interventions to be disseminated and adopted more successfully at each subsequent later stage.

A major strength of this study is that we collected data using several methods and targeting different types of PCPs. Moreover, our study demonstrates high content validity as the questionnaire used in this study is based on qualitative study findings from the same group [15]. However, whereas the samples of the two approaches used (questionnaire, log-data from NHGDoc-server) were quite comparable in terms of background characteristics, selection bias may have occurred with respect to use and attitudes in the self-selected questionnaire. It may well be that responding PCPs used NHGDoc more often and had more positive attitudes towards NHGDoc compared to non-responders. The difference between reported use of NHGDoc in the questionnaire and data from the NHGDoc server points in that direction as well. This should be taken into account in interpreting our findings.

Few thorough process evaluations have been conducted in the field of CDSSs, particularly regarding multiple-domain covering CDSSs implemented at a larger scale. This study used multiple methods, targeting different types of PCPs, and with data collected within the intervention group as well as the control group. Future studies on CDSSs may benefit from thorough evaluations like this one to determine the factors that facilitate or hinder the implementation. Whereas some of the suggested strategies, such as increasing user flexibility, may particularly be helpful in implementing multiple-domain covering CDSSs at a large scale, other strategies, such as involving the end-users in designing the CDSS, also apply to the more common CDSS aimed at a limited number of decision points.

## Conclusions

This process evaluation has identified some factors that might be responsible for the lack of changes in outcomes of the CDSS intervention, as well as useful strategies to improve its implementation. Results indicate that both the CDSS intervention itself as well as its implementation could be improved. Although users are generally positive about CDSSs, a large share of them is insufficiently aware of the functions of NHGDoc and finds the decision support not always useful or relevant and difficult to integrate into daily practice. In designing (similar) CDSS interventions we suggest to more intensely involve the end-users and increase the system’s flexibility and learning capacity. To improve implementation a proper introduction of a CDSS among its target group including adequate training is advocated.

## Additional file

Additional file 1:
**Relevant parts of the questionnaire of the process evaluation of the NHGDoc Evaluation study.** (DOCX 47 kb)

## Abbreviations

CDSS: Computerized decision support system
PCP: Primary care practitioner
GP: General practitioner
PN: Practice nurse
NHG: Dutch College of General Practitioners
EHRS: Electronic health record system
