# Supplementary material for: Exposure to and experiences with a computerized decision support intervention in primary care: results from a process evaluation
Source: BMC Fam Pract. 2015 Oct 16;16:141. doi: 10.1186/s12875-015-0364-0 (PMC4608282; doi:10.1186/s12875-015-0364-0)
Supplement: Additional file 1: — Relevant parts of the questionnaire of the process evaluation of the NHGDoc Evaluation study. (DOCX 47 kb) [file 12875_2015_364_MOESM1_ESM.docx]

**Relevant parts of the questionnaire of the process evaluation of the NHGDoc Evaluation study**

***[Translated into English, originally in Dutch and electronic]***

IQ healthcare, Radboud university medical center

2014

| **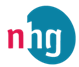** | **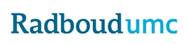** | **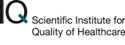** |
| --- | --- | --- |

**Computerized decision support systems**

**in general**

1. Below a number of statements are presented regarding computerized decision support systems in general. Please indicate your level of agreement with each statement, by choosing the response option that applies most.

| 1 | 2 | 3 | 4 | 5 |
| --- | --- | --- | --- | --- |
| Strongly disagree | Disagree | Neither agree nor disagree | Agree | Strongly agree |

| I believe CDSSs are useful sources of advice | 1 | 2 | 3 | 4 | 5 |
| --- | --- | --- | --- | --- | --- |
| I believe CDSSs are useful tools to improve guideline adherence | 1 | 2 | 3 | 4 | 5 |
| I believe that the use of CDSSs results in improved patient care | 1 | 2 | 3 | 4 | 5 |
| I believe CDSSs are relevant for different user groups, such as GPs and PNs | 1 | 2 | 3 | 4 | 5 |
| I fear that the use of CDSSs will lead to ‘cookbook medicine’ | 1 | 2 | 3 | 4 | 5 |
| Overall, I am resistant to using CDSSs | 1 | 2 | 3 | 4 | 5 |
| I lack certain knowledge and/or skills to adequately use CDSS | 1 | 2 | 3 | 4 | 5 |
| I believe that the use of CDSSs is difficult to integrate into daily practice | 1 | 2 | 3 | 4 | 5 |

**The computerized decision support system**

**NHGDoc**

**Knowledge and use of NHGDoc**

1. Below a number of statements are presented regarding your knowledge and use of **NHGDoc.** Pease indicate your level of agreement with each statement, by choosing the response option that applies most.

|  | **Knowledge of NHGDoc** | | **Use of NHGDoc** | | | | |
| --- | --- | --- | --- | --- | --- | --- | --- |
|  | I am aware of the existence/availability | | I use it in practice | | | | |
|  | No | Yes | Never | Rarely | Sometimes | Often | Always |
| The system NHGDoc | **❑** | ❑ | ❑ | ❑ | ❑ | ❑ | ❑ |
| The fact that NHGDoc sends alerts | ❑ | ❑ | ❑ | ❑ | ❑ | ❑ | ❑ |
| The option to ask for/or provide feedback to NHGDoc | ❑ | ❑ | ❑ | ❑ | ❑ | ❑ | ❑ |
| The ‘MyNHGDoc-function’ | ❑ | ❑ | ❑ | ❑ | ❑ | ❑ | ❑ |
| The option to adjust the alerts settings in MyNHGDoc to meet personal needs | ❑ | ❑ | ❑ | ❑ | ❑ | ❑ | ❑ |
| The option to request specific reports in MyNHGDoc regarding the received alerts in the recent past | ❑ | ❑ | ❑ | ❑ | ❑ | ❑ | ❑ |

**Evaluation of the NHGDoc system**

1. Below a number of statements are presented regarding your evaluation/opinion of **NHGDoc**. Please indicate your level of agreement with each statement, by choosing the response option that applies most.

| 1 | 2 | 3 | 4 | 5 | NA |
| --- | --- | --- | --- | --- | --- |
| Strongly disagree | Disagree | Neither agree nor disagree | Agree | Strongly agree | Not applicable |

| *Source and content* |  |  |  |  |  |  |
| --- | --- | --- | --- | --- | --- | --- |
| I believe the source of the NHGDoc content is reliable | 1 | 2 | 3 | 4 | 5 | NA |
| I believe the content of NHGDoc is based on outdated perceptions | 1 | 2 | 3 | 4 | 5 | NA |
| I believe the alert content matches the needs of my profession | 1 | 2 | 3 | 4 | 5 | NA |
| I believe the content of the alerts does not always match with my current needs | 1 | 2 | 3 | 4 | 5 | NA |
| *Format/Lay-out* |  |  |  |  |  |  |
| I believe the appearance of the NHGDoc alert button is too intrusive | 1 | 2 | 3 | 4 | 5 | NA |
| I believe the NHGDoc alert button is sufficiently informative | 1 | 2 | 3 | 4 | 5 | NA |
| I believe the content of the alerts is well readable | 1 | 2 | 3 | 4 | 5 | NA |
| *Functionality* |  |  |  |  |  |  |
| I believe the loading of alerts takes too long | 1 | 2 | 3 | 4 | 5 | NA |
| I believe NHGDoc sends too many alerts | 1 | 2 | 3 | 4 | 5 | NA |
| I believe NHGDoc provides too limited options to adjust the content to my personal needs | 1 | 2 | 3 | 4 | 5 | NA |
| I believe NHGDoc adapts its content to my use of the system and thus provides me with increasingly relevant information | 1 | 2 | 3 | 4 | 5 | NA |

**Interaction of system with daily practice**

| 1 | 2 | 3 | 4 | 5 | NA |
| --- | --- | --- | --- | --- | --- |
| Strongly disagree | Disagree | Neither agree nor disagree | Agree | Strongly agree | Not applicable |

| Using NHGDoc has a negative impact on doctor-patient communication | 1 | 2 | 3 | 4 | 5 | NA |
| --- | --- | --- | --- | --- | --- | --- |
| The alert content does not always match the patient’s current reason for visiting | 1 | 2 | 3 | 4 | 5 | NA |
| Using NHGDoc does not take much extra time | 1 | 2 | 3 | 4 | 5 | NA |
| Using NHGDoc requires too much additional work (during or after consultation) | 1 | 2 | 3 | 4 | 5 | NA |
| NHGDoc is sufficiently integrated with other systems (electronic prescribing system, printing patient letters etc.) | 1 | 2 | 3 | 4 | 5 | NA |
| I fear that using NHGDoc results in misuse of patient data/medical practice data by third parties (such as insurance companies). | 1 | 2 | 3 | 4 | 5 | NA |

1. Do you have any idea on which clinical topic this study is focused on?
